# Supplementary material for: Mechanistic prediction of community composition across resource conditions and species richness
Source: Nat Commun. 2025 Nov 12;16:9962. doi: 10.1038/s41467-025-64935-5 (PMC12612213; doi:10.1038/s41467-025-64935-5)
Supplement: Supplementary file 2 — Reporting Summary [file 41467_2025_64935_MOESM2_ESM.pdf]

nature portfolio

Corresponding author(s): Zhijie Zhang

Last updated by author(s): Sep 16, 2024

Reporting Summary

Nature Portfolio wishes to improve the reproducibility of the work that we publish. This form provides structure for consistency and transparency in reporting. For further information on Nature Portfolio policies, see our Editorial Policies and the Editorial Policy Checklist.

Statistics

For all statistical analyses, confirm that the following items are present in the figure legend, table legend, main text, or Methods section.

n/a	Confirmed	
☐	☒	The exact sample size (n) for each experimental group/condition, given as a discrete number and unit of measurement
☐	☒	A statement on whether measurements were taken from distinct samples or whether the same sample was measured repeatedly
☐	☒	The statistical test(s) used AND whether they are one- or two-sided<br>*Only common tests should be described solely by name; describe more complex techniques in the Methods section.*
☒	☐	A description of all covariates tested
☐	☒	A description of any assumptions or corrections, such as tests of normality and adjustment for multiple comparisons
☐	☒	A full description of the statistical parameters including central tendency (e.g. means) or other basic estimates (e.g. regression coefficient) AND variation (e.g. standard deviation) or associated estimates of uncertainty (e.g. confidence intervals)
☐	☒	For null hypothesis testing, the test statistic (e.g. F, t, r) with confidence intervals, effect sizes, degrees of freedom and P value noted<br>*Give P values as exact values whenever suitable.*
☐	☒	For Bayesian analysis, information on the choice of priors and Markov chain Monte Carlo settings
☐	☒	For hierarchical and complex designs, identification of the appropriate level for tests and full reporting of outcomes
☒	☐	Estimates of effect sizes (e.g. Cohen's d, Pearson's r), indicating how they were calculated

Our web collection on statistics for biologists contains articles on many of the points above.

Software and code

Policy information about availability of computer code

Data collection	Data collection was done in R (version. 4.2.3). All data has been deposited in Figshare.
Data analysis	The analyses were done in R (version. 4.2.3) and python (version. 3.8.10). Code examples are provided during the peer review. All code has been deposited to Figshare.

For manuscripts utilizing custom algorithms or software that are central to the research but not yet described in published literature, software must be made available to editors and reviewers. We strongly encourage code deposition in a community repository (e.g. GitHub). See the Nature Portfolio guidelines for submitting code & software for further information.

Data

Policy information about availability of data

All manuscripts must include a data availability statement. This statement should provide the following information, where applicable:

- Accession codes, unique identifiers, or web links for publicly available datasets
- A description of any restrictions on data availability
- For clinical datasets or third party data, please ensure that the statement adheres to our policy

All code and data will be upload to Figshare if the manuscript is accepted.

Research involving human participants, their data, or biological material

Policy information about studies with human participants or human data. See also policy information about sex, gender (identity/presentation), and sexual orientation and race, ethnicity and racism.

Reporting on sex and gender	The research does not involve human participants.
Reporting on race, ethnicity, or other socially relevant groupings	The research does not involve human participants.
Population characteristics	The research does not involve human participants.
Recruitment	The research does not involve human participants.
Ethics oversight	The research does not involve human participants.

Note that full information on the approval of the study protocol must also be provided in the manuscript.

Field-specific reporting

Please select the one below that is the best fit for your research. If you are not sure, read the appropriate sections before making your selection.

☐ Life sciences ☐ Behavioural & social sciences ☒ Ecological, evolutionary & environmental sciences

For a reference copy of the document with all sections, see nature.com/documents/nr-reporting-summary-flat.pdf

Ecological, evolutionary & environmental sciences study design

All studies must disclose on these points even when the disclosure is negative.

Study description	We tracked the daily growth rates of 12 different phytoplankton species (green algae; Supplement S1) in monocultures over four days (approx. zero to eight generations, depending on the resource concentrations). This was conducted under 12 concentrations of nitrate (NO3–N), ammonium (NH4–N), or phosphorus (P; Fig. 1b). With two replicates, this totaled 864 cultures. Next, we grew two, three, four, or six species in semi-continuous cultures where they competed for different ratios of two essential resources (NO3–N and P) or of two substitutable resources (NO3-N and NH4–N; Figs. S1, 2). We tracked the community composition over 12 days. With two replicates, this totaled 960 cultures.
Research sample	We sampled each culture 20 ul, because our pilot experiment showed that such volume contained 10 to 1000 cells, which is ideal for counting and identifying species.
Sampling strategy	All sampling was done by a pipetting robot (OT-2, Opentrons).
Data collection	All samples were imaged and counted with a high-content microscopy (ImageXpress® Micro 4 High-Content Imaging System).
Timing and spatial scale	The monoculture experiment started on Jan 31 and stopped on Feb 5, 2024, and was sampled every day from Feb 1 to 4. The competition experiment started on Jan 31 and stopped on Feb 12, 2024, and was sampled on Feb1 and every two days from Feb 4 to 12. We sampled less frequently in the competition experiment because it last longer and had a larger sample size than the monoculture experiment. The spatial scale is local.
Data exclusions	For algal communities where fewer than 10 cells were identified, we excluded the data points. This aims to minimize the effect of randomness on cell sampling and counting.
Reproducibility	Note that the experiment contained two replicates for each species/community per treatment. The two replicates showed similar results.
Randomization	The species and treatments were randomized during the experiment.
Blinding	The data was collected by machines and code, ensuring that no human bias was introduced.

Did the study involve field work? ☐ Yes ☒ No

Reporting for specific materials, systems and methods

We require information from authors about some types of materials, experimental systems and methods used in many studies. Here, indicate whether each material, system or method listed is relevant to your study. If you are not sure if a list item applies to your research, read the appropriate section before selecting a response.

Materials & experimental systems

n/a	Involved in the study
☒	☐ Antibodies
☒	☐ Eukaryotic cell lines
☒	☐ Palaeontology and archaeology
☒	☐ Animals and other organisms
☒	☐ Clinical data
☒	☐ Dual use research of concern
☒	☐ Plants

Methods

n/a	Involved in the study
☒	☐ ChIP-seq
☒	☐ Flow cytometry
☒	☐ MRI-based neuroimaging

Plants

Seed stocks	The algae used in the experiment were from the algae collection at the Limnological Institute of the University of Konstanz
Novel plant genotypes	The research did not use any novel plant genotypes.
Authentication	The algae were authenticated for scientific research.
